# Synthesis of Isomers of Eugenol^1^

**DOI:** 10.6028/jres.067A.026

**Published:** 1963-06-01

**Authors:** Gerhard M. Brauer, Richard Warren Morris, Willard B. Howe

## Abstract

In connection with an investigation of the correlation between structure and reactivity of eugenol isomers, *o*-eugenol, 3-allyl-2-methoxyphenol and chavibetol were synthesized. A five-step synthesis was found to be most suitable for the preparation of 3-allyl-2-methoxyphenol. An improved separation of chavibetol from eugenol was achieved by gas chromatography.

## 1. Introduction

Slurries of zinc oxide and eugenol (I) harden to form a cementitious product which has found many applications in dentistry. A prior investigation of the setting mechanism showed that the hardened mass consists of zinc oxide embedded in a matrix of chelated zinc eugenolate [l].^3^ It therefore appeared of interest to study the properties and reactivity of all eugenol isomers capable of forming chelates with metal oxides, i.e., *o*-eugenol (II), 3-allyl-2-methoxyphenol (III) chavibetol (IV) and their respective propenyl isomers.

**Figure:**
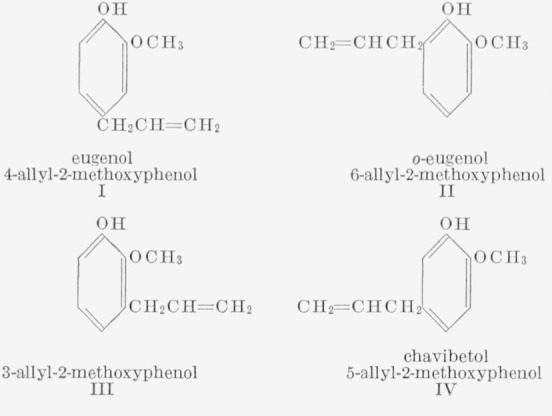


The present paper describes methods for synthesizing some of these compounds. In a subsequent paper the structure of the isomers will be correlated with their ultraviolet and infrared absorption spectra, ionization constants and reactivity with metal oxides.

**Figure 1:**
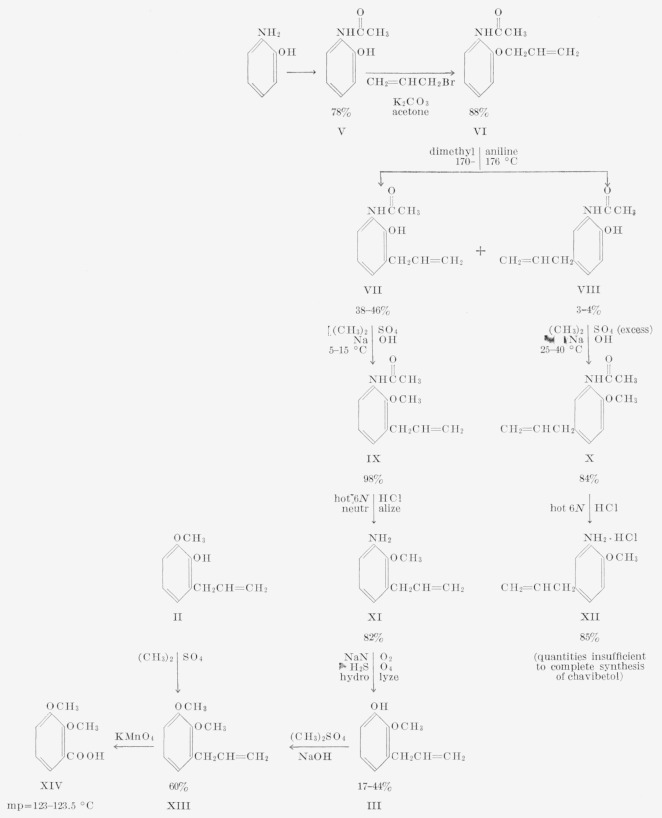
Synthesis of 3-allyl-2-methoxyphenol (III).

Three procedures were considered for the synthesis of 3-allyl-2-methoxyphenol (III). (1) Partial demethylation of l-allyl-2,3-dimethoxybenzene (a preparation analogous to that described by Hirao [2] and Schöpf and coworkers [3] for the synthesis of Chavibetol). The isomeric products II and III appeared to be difficult to separate by techniques other than gas chromatography. (2) Claisen rearrangement of allyl 2-benzoyloxyphenyl ether followed by methylation and by subsequent hydrolysis of the benzoyl group. However, the rearrangement gave 1-allyl- 2,3-dihydroxybenzene instead of the desired intermediate. (3) The procedure outlined in figure 1; this synthesis proved to be successful.^4^

The preparation of IV was also attempted by several methods. The synthesis of pure chavibetol described by Hirao [2] and Schöpf and coworkers [3] proved to be laborious in the separation of chavibeto from its eugenol isomer. This separation has now been greatly facilitated by the use of gas chromatography.

## 2. Experimental Procedures

### 2.1. o-Eugenol (6-Allylguaiacol)^5^ (II) and o-Eugenol Benzoate

**Figure 2:**
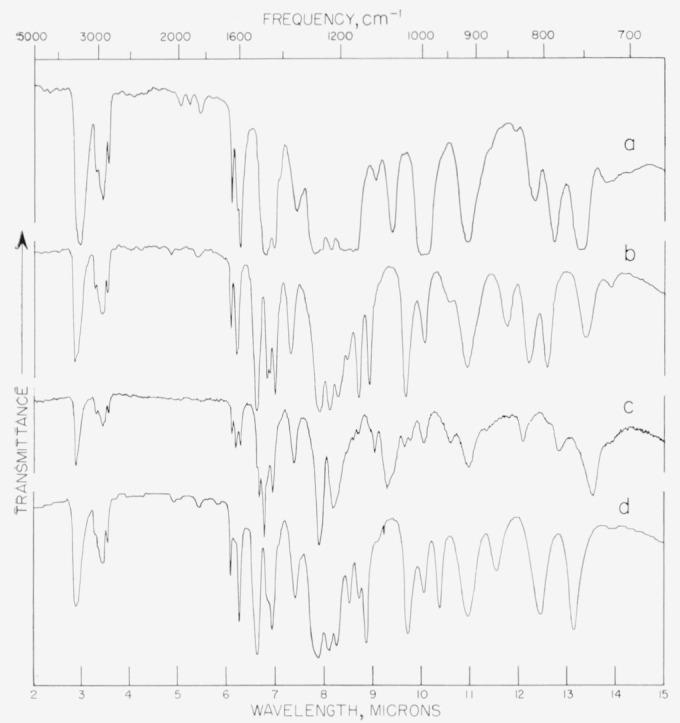
Infrared absorption curves of eugenol and its isomers. Top to bottom: (a) 3-allyl-2 methoxyphenol, (b) eugenol, (c) o-eugenol, (d) havibetol.

*o*-Eugenol was synthesized from guaiacol in 67-percent yield by the procedure of Allen and Gates [5]. The infrared spectrum is given in figure 2. *o*-Eugenol benzoate was prepared by refluxing *o*-eugenol with benzoyl chloride. The solution was poured into cold water and the mixture extracted with ether. The ether solution was washed successively with dilute NaOH and HCl and evaporated. After recrystallizations from aqueous ethanol and from ether the solid melted at 59 to 60 °C.

*Anal:* Calcd. for C_17_H_16_O_3_: C, 76.1; H, 6.0. Found: C, 76.6; H, 6.0.

### 2.2. 3-Allyl-2-methoxyphenol (3-Allylguaiacol) (III)

#### o-Hydroxyacetanilide (2′-Hydroxyacetanilide) (V)

This compound was synthesized from 283 g (2.59 mole) of *o*-hydroxyaniline according to the procedure of Fierz-David and Kuster [6] and recrystallized from acetone. Yield: 307 g (78 percent).

#### o-Hydroxyacetanilide Allyl Ether (2′-Hydroxyacetanilide Allyl Ether) (VI)

This compound was synthesized as described by Tiffany [7]. Yield: 88 percent.

#### 3-Allyl-2-hydroxyacetanilide (3′-Allyl-2′-hydroxyacetanilide) (VII) and 5-Allyl-2-hydroxyacetanilide (5′- Allyl-2′-hydroxyacetanilide) (VIII)

The Claisen rearrangement of VI was conducted in dimethylaniline at 170 to 176 °C [7]; time of reflux varied from 1½ to 4 hr. The two isomers were fractionally recrystallized and separated as described previously [7]. Faster separation was obtained by repeated fractional precipitation from 1 N NaOH by the addition of 0.5 N H_2_S0_4_ [8]. Yield of VII, 38 to 46 percent, mp 114 to 115 °C; yield of VIII, 3 to 4 percent, mp 115 to 116 °C. The purity of the isomers was determined by mixed melting points.

**Figure 3:**
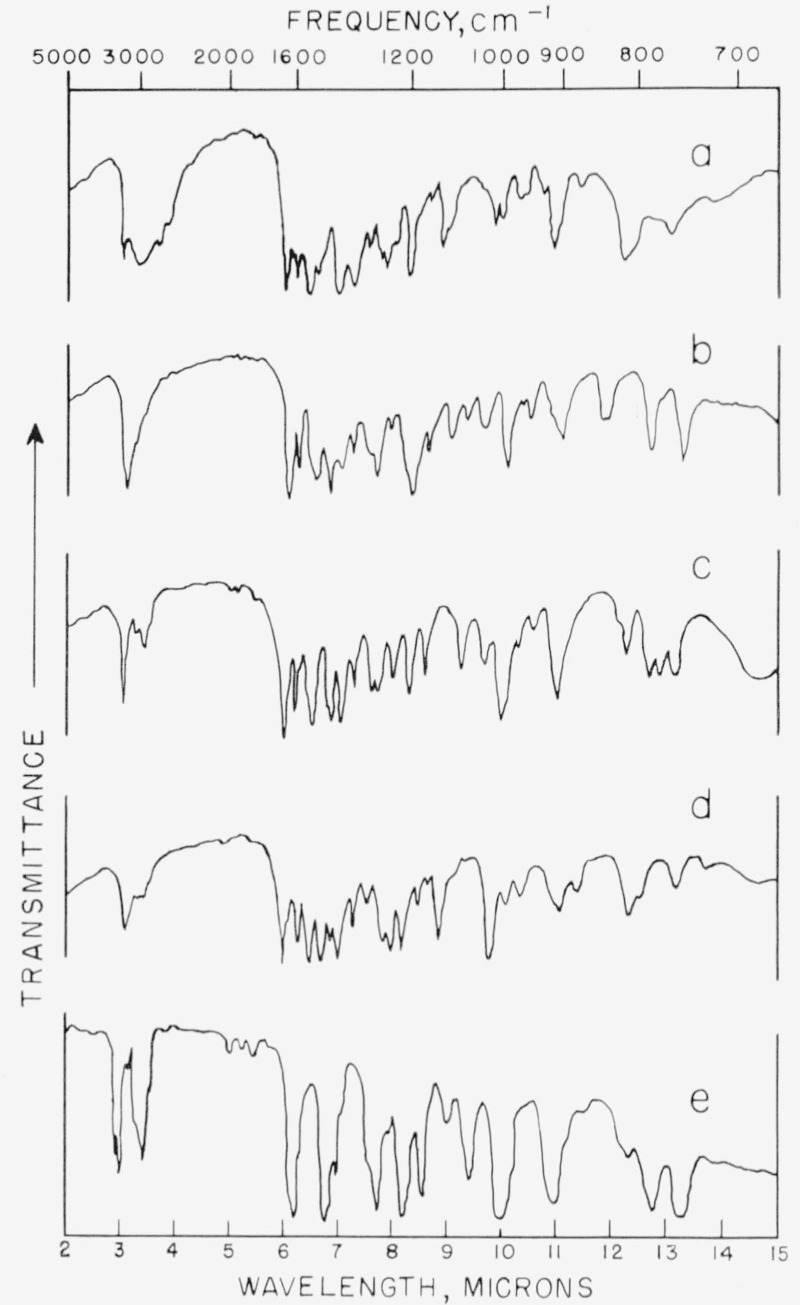
Infrared absorption curves of intermediates. Top to bottom: (a) 3-allyl-2-hydroxyacetanilide, (b) 5-allyl-2-hydroxyaeetanilide, (c) 3-allyl-2-methoxyacetanilide, (d) 5-allyl-2-mcthoxyacetanilide, (e) 3-allyl-2-methoxyaniline. Materials (a) through (d) were in the form of potassium iodide pellets; material (e) was a liquid.

The infrared spectra of the isomers are shown in figures 3a and 3b. The spectra are useful in detecting isomerization of the allyl group to the propenyl, a change which takes place readily. Presence of the allyl group is indicated by the out-of-plane vibrations of the =CH_2_ and =CH— groups which give rise to two strong absorptions, one between 9.97 and 10.12 *μ* and the other between 10.93 to 11.09 *μ* [9]. Intramolecular hydrogen bonding between the phenolic hydrogen and the carbonyl is indicated by the shift of the acetamido carbonyl absorption (usually occuring near 5.99 *μ*) to 6.09 *μ*.

#### 3-Allyl-2-methoxyacetanilide (3′-Allyl-o-acetanisidide) (IX)

3-Allyl-2-hydroxyacetanilide (156 g, 0.82 mole) dissolved in 500 ml of water containing 39.2 g (0.98 mole) of NaOH was cooled to 5 °C, and nitrogen was passed over the solution. Dimethyl sulfate (100 ml, 1.055 moles) was gradually added during 1 hr to the stirred solution, which was kept at 5 to 12 °C. Additional dimethyl sulfate (30 ml, 0.32 mole) was added during a period of 2½ hr. The reaction mixture, warmed to room temperature, was made alkaline and extracted with ether. On evaporation of the extract and crystallization of the residue from ligroin or 50-percent aqueous ethanol, colorless platelets were obtained. Yield: 163 g (98 percent), mp 86 to 87 °C.

*Anal:* Calcd. for C_12_H_15_NO_2_: C, 70.3; H, 7.4; N, 6.8. Found: C, 70.4; H, 7.2; N, 6.9.

The infrared spectrum is given in figure 3c.

#### 3-Allyl-2-methoxyaniline (3′-Allyl-o-anisidine) (XI) and its Hydrochloride

3-Allyl-2-methoxyacetanilide (46.6 g, 0.23 mole) was refluxed for 80 min with 45 ml of 6 *N* HCl. The solution was made alkaline with 10-percent NaOH, and the oily layer was separated. Ether extracts of the aqueous layer were added to the brown oil. After evaporation of the ether the remaining oil was distilled at 3.5 mm in the presence of a little hydroquinone (to minimize polymer formation). The liquid turned brown on standing in air. Yield: 30.3 g (82%). For further purification some of the product was fractionated in a Piros-Glover spinning-band still (bp 78 to 80 °C at less than 1 mm); $n_{D}^{25}=1.553$.

*Anal:* Calcd. for C_10_H_13_NO: C, 73.6; H, 8.0; N, 8.6. Found: C, 73.5; H, 8.0 ; N, 8.3.

The absence of characteristic infrared absorption bands (fig. 3c) for *cis* and *trans* — CH = CH— indicates that little, if any, of the propenyl isomer was present.

The hydrochloride was prepared by dissolving 1 g of XI in 15 ml of ether and bubbling hydrogen chloride through the solution for 5 min. The white solid, obtained on recrystallization from hot ethanol containing a drop of concentrated HCl, melted with decomposition at 178 to 179 °C.

*Anal:* Calcd. for C_10_H_14_ClNO: C, 60.2; H, 7.1; N, 7.0. Found: C, 59.9; H, 7.4; N, 6.9.

#### 3-Allyl-2-methoxyphenol (3-Allylguaiacol) (III)

3-Allyl-2-methoxyaniline was diazotized, and the resulting diazonium salt converted to the phenol. Reverse diazotization appeared to give slightly better yields than the usual addition of sulfuric acid followed by nitrite. Yields improved when only small quantities of diazonium salt were decomposed; however, they varied considerably from one preparation to another.

3-AlIyl-2-methoxy aniline (5 g, 0.03 mole) was suspended in 25 ml of water, and solutions of 1.15 g of NaOH in 6.5 ml of water and of 4 g of NaN0_2_ in 15 ml of water were added successively. The entire solution, cooled to 0 °C, was then quickly added, with stirring, to 4.5 g of concentrated H_2_SO_4_ (previously cooled to 0 °C) and kept in an ice bath. The excess nitrite was decomposed by the addition of urea until a negative test for nitrite was obtained with starch-iodide paper. The resulting cold solution was stored in a refrigerator until needed.

A solution prepared from 150 g of anhydrous Na_2_SO_4_, 108 ml of concentrated H_2_SO_4_ and 100 ml of water, in a three-necked flask equipped for steam distillation, was kept at 135 to 150 °C by a surrounding oil bath. The cold solution of the diazonium salt was slowly added from a separatory funnel while steam was passed through the flask; the volatile products were thus steam-distilled immediately after decomposition of the diazonium salt.^6^ The distillate was extracted with ether, the ether extracts were washed with 10-percent, aqueous NaHCO_3_, and the phenol was extracted from the ether layer with 10- percent aqueous NaOH. After ether extraction of the acidified solution and evaporation of the solvent 0.85 to 2.2 g (17 to 44%) of 3-allyl-2-methoxyphenol was obtained; bp 92 to 93 °C/4 mm, $n_{D}^{26}=1.535$.

*Anal:* Calcd. for C_10_H_12_O_2_: C, 73.1; H, 7.4. Found: C, 72.9; H, 7.3.

The infrared spectrum (fig. 2) shows absorption bands in the regions in which the —CH=CH_2_ group absorbs, namely, 5.39 to 5.55 *μ*; 7.04 to 7.09 *μ*; 7.69 to 7.76 *μ*; 10.05 to 10.15 *μ*; and 10.93 to 11.05 *μ.* [11]. The areas in which the *trans* —CH = CH— group absorbs (7.67 to 7.72 *μ* and 10.31 to 10.42 *μ*) fall on the sides of two of these bands, and hence it is not possible to state with certainty that there is no absorption due to *trans* —CH = CH— groups. Absence of an absorption band near 14.49 *μ* indicates that there is very little, if any, *cis —*CH=CH— present. Before the final fractionation the phenol contained a considerable amount of ethanol. The mechanism of the formation of this product is not known.

To establish the identity of III it was converted to 2,3-dimethoxybenzoic acid (*o*-veratric acid) (XIV) via l-allyl-2,3-dimethoxybenzene (XIII). 3-Allyl-2-methoxyphenol (1.5 g, 0.09 mole) dissolved in 16 ml of 15-percent NaOH was cooled, and 2.0 ml of dimethyl sulfate was rapidly added to the stirred solution. After 15 min 2 ml of dimethyl sulfate was added, and the solution was refluxed for 2 hr; 5.5 ml of 10-percent NaOH was then added, the solution refluxed for 2 more hr, cooled, and extracted with ether. The ether extract was dried and evaporated, and the residue distilled at 65 to 68 °C/2.5 mm. Yield: 1.0 g (60 percent), $n_{D}^{25}=1.524$. The same physical constants were obtained for XIII, synthesized from *o*-eugenol according to the procedure of Mauther [12].

For the conversion of XIII to XIV, 1 g of 1-allyl-2,3-dimethoxybenzene was refluxed for 1½ hr with 3.6 g of powdered potassium permanganate dissolved in 40 ml of water. The mixture was filtered through a fritted glass funnel, and the filtrate was evaporated to about 20 ml, cooled, and acidified with dilute H_2_SO_4_. The resulting precipitate, after successive recrystallizations from benzene and water, melted at 123 to 123.5 °C. The melting point was unchanged by mixture with authentic 2,3-dimethoxybenzoic acid.

#### Conversion of III to 2-Methoxy-3-propenylphenol

3-Allyl-2-methoxyphenol (III) was refluxed with alcoholic KOH for 16 hr by a method analogous to the preparation of isochavibetol [2]; bp 109 °C/7mm, $n_{D}^{25}=1.556$. As indicated by the infrared spectrum the product was an impure mixture of *cis* and *trans* 2-methoxy-3-propenylphenol.

### 2.3. Chavibetol (5-Allylguaiacol) (IV)

Eugenol methyl ether (l-allyl-3,4-dimethoxybenzene) was synthesized in 88-percent yield according to the procedure of Luff, Perkin, and Robinson [13]. Demethylation of this compound with Grignard reagent, conducted as suggested by Schöpf and coworkers [3] gave a mixture of eugenol and chavibetol.

It was found that these impure eugenolchavibetol mixtures could be separated by gas chromatography with a diisodecyl phthalate column heated to 175 °C and a hydrogen flame detector. Larger quantities of the isomers were most conveniently separated by a chromatograph designed for synthetic work; this employed Apiezon J on a C-22 firebrick column, with a column temperature of 180 °C and a pressure of 10 lb/in^2^. The infrared spectrum of chavibetol is shown in figure 2.

The chavibetol was characterized by conversion to isochavibetol (mp 95 to 96 °C) as described by Hirao [2].

### 2.4. Preparation of Intermediates in Attempted Synthesis of Chavibetol

Prior to the gas chromatographic separation described in section 2.3, the preparation of chavibetol from VIII (fig. 1) was attempted. Although the preparation was not completed for lack of material, two new intermediates (X and XII) were obtained. The following sections describe their preparation.

#### 5 - Allyl - 2 - methoxyacetanilide (5 - Allyl - o - acet - anisidide) (X)

2-Acetamido-4-allylphenol (5 g, 0.026 mole) was dissolved in 25 ml of 5.5-percent aqueous NaOH (0.034 mole). During a 15-min period, 3.8 ml (0.04 mole) of dimethyl sulfate was added, while the solution was stirred in an atmosphere of nitrogen. During 90 min, 3 ml of dimethyl sulfate was added, together with sufficient NaOH solution to keep the mixture alkaline. The mixture was then heated at 40 °C for 45 min, and extracted with ether. After drying and evaporation of the solvent, the residue was recrystallized from ligroin to yield long, colorless needles, mp 56.5 to 57 °C; yield; 4.50 g (84 percent).

*Anal:* Calcd. for C_12_H_16_NO_2_: C, 70.3; H, 7.4; N, 6.8. Found: C, 70.3; H, 7.6; N, 6.5. The infrared spectrum is given in figure 3d.

#### 5-Allyl-2-methoxyaniline Hydrochloride (5′-Allyl- o-anisidine Hydrochloride) (XII)

5-Allyl-2-methoxyacetanilide (3.8 g, 0.019 mole) dissolved in 10 ml of 6*N* HC1 was refluxed for 45 min, and the solution was concentrated by evaporation under reduced pressure. The resulting precipitate on recrystallization from an aqueous solution acidified with a little HCl gave colorless needles (mp 170 to 172 °C); yield (including recovery from mother liquor); 3.15 g (85 percent).

*Anal:* Calcd. for C_10_H_14_ClNO: C, 60.2; H, 7.1; N, 7.0. Found: C, 60.2; H, 7.2; N, 6.8.

A solution of the hydrochloride (3 g in 15 ml of water) was made slightly alkaline with 2-percent NaOH and extracted with ether. After evaporation of the solvent a liquid remained which sublimed during attempted vacuum distillation. The crystalline product, on successive recrystallization from 70-percent aqueous ethanol and ligroin, gave colorless scales, mp 47 °C. The presence of an absorption band at 10.32 *μ* suggests that some isomerization to the *trans* propenyl group had taken place. Elementary analysis indicated that the compound was impure.

### 2.5. Synthesis of Allyl 2-Benzoyloxyphenyl Ether and Its Behavior in a Claisen Rearrangement

In one of the alternative procedures for the synthesis of 3-allyl-2-methoxyphenol, allyl 2-benzoyloxyphenyl ether was synthesized and its Claisen rearrangement investigated.

#### Allyl 2-Benzoyloxyphenyl Ether (XV)

Catechol monobenzoate was prepared in 55-percent yield by benzoylation of catechol at 6 °C according to the procedure of Donadio [14]. A solution prepared from catechol monobenzoate (168 g, 0.78 mole), anhydrous K_2_CO_3_ (110 g, 0.80 mole), allyl bromide (103 g, 0.85 mole) and 750 ml of dry acetone was refluxed overnight with stirring. The product was extracted with ether, and the extract washed successively with 5-percent NaOH and water. After evaporation of the solvent the compound was recrystallized from an ether-petroleum ether mixture ; yield ; 121 g (61 %) ; mp 44 °C.

*Anal*: Calcd. for C_16_H_14_O_3_: C, 75.6; H, 5.6. Found: C, 75.6; H, 5.7.

#### Behavior of Allyl 2-Benzoyloxyphenyl Ether on Claisen Rearrangement

Attempts were made to obtain 2-allyl-6-benzoyloxyphenol by Claisen rearrangement of allyl 2-benzoyloxyphenyl ether. The allyl ether (10 g, 0.04 mole) was refluxed at 180 to 200 °C in a nitrogen atmosphere for 30 min.

Separation of the reaction products yielded considerable quantities of starting materials, benzoic acid, and a phenol which distilled at 107 to 115 °C/7mm. The distillate $\left(n_{D}^{26}=1.5588\right)$ crystallized on storage in a refrigerator; yield: 2.2 g (37%).

The phenol was identified as impure 1-allyl-2,3-dihydroxybenzene by (1) its infrared spectrum, which showed the presence of allyl and phenol groups and the absence of ester linkages, (2) a positive test for a catechol derivative [15], and (3) methylation with dimethyl sulfate and subsequent permanganate oxidation to 2,3-dimethoxybenzoic acid.

## Footnotes

3 Figures in brackets indicate the literature references at the end of this paper.

4 Since completion of this work, the preparation of III from *o*-nitrophenol [4] has come to our attention.

5 Throughout this paper the systematic names employed by *Chemical Abstracts* are given parenthetically at the first mention of the compound in the experimental section.

6 Because of tar formation, the solution of the diazonium salts was introduced, in later reactions, through a cold-finger addition tube (as suggested by Lambooy [10]); the tube reached to within 1 cm of the surface of the hydroysis mixture.
